# Anhedonia Relates to the Altered Global and Local Grey Matter Network Properties in Schizophrenia

**DOI:** 10.3390/jcm10071395

**Published:** 2021-03-31

**Authors:** Byung-Hoon Kim, Hesun Erin Kim, Jung Suk Lee, Jae-Jin Kim

**Affiliations:** 1 Department of Psychiatry, Yonsei University College of Medicine, Seoul 03722, Korea; egyptdj@yonsei.ac.kr; 2 Institute of Behavioral Science in Medicine, Yonsei University College of Medicine, Seoul 03722, Korea; erinkim791@yuhs.ac (H.E.K.); thanato96@naver.com (J.S.L.); 3 Department of Psychiatry, National Health Insurance Service Ilsan Hospital, Goyang, Gyeonggi-do 10444, Korea; 4 Department of Psychiatry, Gangnam Severance Hospital, Yonsei University College of Medicine, 211 Eonju-ro, Gangnam-gu, Seoul 06273, Korea

**Keywords:** schizophrenia, anhedonia, grey matter network, graph theory

## Abstract

Anhedonia is one of the major negative symptoms in schizophrenia and defined as the loss of hedonic experience to various stimuli in real life. Although structural magnetic resonance imaging has provided a deeper understanding of anhedonia-related abnormalities in schizophrenia, network analysis of the grey matter focusing on this symptom is lacking. In this study, single-subject grey matter networks were constructed in 123 patients with schizophrenia and 160 healthy controls. The small-world property of the grey matter network and its correlations with the level of physical and social anhedonia were evaluated using graph theory analysis. In the global scale whole-brain analysis, the patients showed reduced small-world property of the grey matter network. The local-scale analysis further revealed reduced small-world property in the default mode network, salience/ventral attention network, and visual network. The regional-level analysis showed an altered relationship between the small-world properties and the social anhedonia scale scores in the cerebellar lobule in patients with schizophrenia. These results indicate that anhedonia in schizophrenia may be related to abnormalities in the grey matter network at both the global whole-brain scale and local–regional scale.

## 1. Introduction

Schizophrenia includes diverse symptoms, such as delusions, hallucinations, disorganized speech, disorganized behavior, and negative symptoms [1]. Among these symptoms, negative symptoms are characterized by the absence of certain psychological features, including affective experience, motivation, and verbal or nonverbal expression [2,3]. The long-term prognosis of schizophrenia is dependent on how well the negative symptoms are controlled [4,5]. However, not much is known about the neurological pathophysiology of negative symptoms, and not many options are available for treating the negative symptoms [6].

One of the negative symptoms in schizophrenia is anhedonia, which is defined as the loss of hedonic experience or pleasure to various stimuli in real life [7]. The broad definition of anhedonia may include reduced experiences of both positive and negative emotions, but usually, positive emotion is thought to be diminished in schizophrenia [8]. The pathophysiology underlying anhedonia has been a topic of interest for clinical neuroscientists. Neural correlates that contribute to the emergence of anhedonia are diverse and complex [9]. In particular, previous studies have consistently reported abnormalities in the reward system, such as the orbitofrontal cortex, anterior cingulate cortex (ACC), ventral tegmental area, striatum, hippocampus, and amygdala [10,11]. Impairment of the neural networks related to anticipation, emotion processing, salience processing, and attention to the expected or given rewards can all result in the inability to feel pleasure [12,13,14,15].

There are two types of anhedonia that patients may suffer from—physical and social anhedonia [7,16]. The focus of this study was to investigate the relationship between these two types of anhedonia and brain grey matter (GM) network properties in schizophrenia. Alterations of the GM have been studied using structural magnetic resonance imaging (MRI), and one of the most widely applied methods in these studies is voxel-based morphometry, which has revealed changes in the regional GM volumes in patients with schizophrenia [17]. Using this methodology for anhedonia, there is a report that a number of regional volumes show a different trend of correlations with the level of physical anhedonia between patients with schizophrenia and healthy controls [18]. Another way to study GM abnormality is surface-based morphometry (SBM), which evaluates the surface properties such as the area, curvature, and thickness of the brain surface. The SBM in patients with first-episode schizophrenia revealed aberrant surface morphology in the ACC [19]. A study evaluating the cortical gyrification in patients with schizophrenia found abnormalities in the parahippocampal gyrus and lingual gyrus [20].

Analysis of morphological covariation of the GM is another option for evaluating the GM abnormality, in which the construction of single-subject individual networks based on the similarity of the cortical thickness and folding of the GM is performed. The constructed single-subject GM networks are then evaluated with the graph-theoretical network properties, such as path length, clustering coefficient, and small worldness [21]. The GM networks have been found to be disrupted in schizophrenia and other psychotic disorders [22,23], but their relationship with specific symptoms of schizophrenia including anhedonia remains to be clarified.

The topology of the brain in schizophrenia is characterized by the reduced small-world property of structural and functional networks defined from MRI volumes [24]. The small-world property of a network is defined as its relative clustering/path length ratio with respect to a random network [25]. It can be said that a network with more small worldness is more likely to contain high degree vertices serving as a hub of the network, leading to a shorter path length between two nodes within the network [26]. It is thought that decreased small worldness represents the inefficient topology of functional networks in schizophrenia, prompting more severe symptoms of the illness [24,27]. Previous studies that analyzed the diffusion tensor imaging (DTI) in a graph theoretical way found both globally and locally disrupted small worldness of the brain structural connectivity network in schizophrenia [28,29]. The disruption was found to be associated with the longitudinal clinical symptoms of schizophrenia [30]. The reduction of small-world property of the functional networks from the resting-state functional MRI has also been reported consistently [24]. This alteration of functional network topology is potentially related to clinical application in that it may reflect the symptom severity or the treatment response in schizophrenia [31]. There is evidence that the topological property of a structural GM network, which is of our interest, is closely related to that of a functional network [32,33]. While structural and functional networks reflect correlations between regional activities, the GM networks represent the covariance pattern of the cortical structure.

This study aimed to obtain new insight for understanding the neural mechanism of anhedonia in schizophrenia using an approach for constructing the GM network and assessing the global scale network topology of the whole brain. Based on previous findings, we hypothesized that the single-subject GM network of patients with schizophrenia would exhibit decreased small worldness in the GM network, compared to healthy controls, and that such a decrease would be related to the severity of anhedonia. The second endpoint of this study was to investigate the GM network’s small worldness at a local scale, which was expected to reveal brain regions with GM network abnormality that underlie anhedonia in schizophrenia. To analyze the GM network’s small worldness at the local scale, we constructed the single-subject network from the brain regions based on two standard space atlases. The single-subject GM networks were constructed from the T1-weighted MRI images and were statistically analyzed to test these hypotheses.

## 2. Methods

### 2.1. Subject Demographics and Scale Measurements

There were 123 subjects diagnosed with schizophrenia in the patient group and 160 healthy adults in the control group. The diagnostic procedure for inclusion to the patient group and exclusion to the control group was made using the Structured Clinical Interview for the Diagnostic and Statistical Manual of Mental Disorders, fourth edition (SCID) [34]. Exclusion criteria were the presence of significant neurological, medical or psychiatric illness other than schizophrenia, current or past substance abuse, and contraindication for the MRI scanning. The duration of illness and the chlorpromazine-equivalent dosage of current antipsychotic medication were assessed in the patient group. This study was approved by the Institutional Review Board of Yonsei University Severance Hospital (4-2015-0148). Informed consent was obtained from all participating subjects.

To measure the level of anhedonia in both groups, the Chapman physical anhedonia scale (PAS) and social anhedonia scale (SAS) were collected from the subjects [35]. The PAS and SAS consist of 61 and 40 questions with True–False responses measuring how much pleasure one can experience from physical and social activities and yielding scores ranging from 0 to 61 and 0 to 40, respectively. While the PAS data were obtained from all subjects, the SAS was measured only in 77 in the patient group and 104 in the control group.

### 2.2. Image Acquisition and Anatomical Processing

The MRI scanning was conducted with a 3.0 Tesla MRI scanner (Intera Achieva; Philips Medical Systems, Best, the Netherlands). High resolution horizontal images were acquired with a T1-weighted gradient-echo sequence (echo time = 4.6 ms; repetition time = 9.7 ms; flip angle = 30°; field of view = 220 mm; number of slices = 180; and image matrix = 256 × 256). Anatomical processing of the images was performed with the FSL v6.0.1 [36]. The images were first cropped to the adequate field of view, followed by skull stripping and brain extraction. The linear and nonlinear warp field for registration to the standard Montreal Neurological Institute (MNI) space was computed at this stage since the inverse of the warp field was later used for producing the subject-specific masks from the standard space atlases. Tissue type segmentation was conducted with the FAST package [37], resulting in partial volume segmentations of the GM, white matter, and cerebrospinal fluid. Segmented GM images were visually inspected for validity, and two subjects from the patient group were excluded due to invalid anatomical processing results. For the valid GM images, the total GM volume was calculated with the *fslstats* function of the FSL.

### 2.3. Construction of the Networks and Measurement of the Network Small-World Property

The images were first assured to be resliced to isotropic 2 mm voxels, and the cube of 3 × 3 × 3 voxels served as a node of the graph. There were 6820 nodes on average with a standard deviation of 775.1 in the network graph. The method for constructing the similarity matrix, which serves as the edges of the network graph, from the defined nodes was based on the original work of the similarity-based extraction method [21]. For binarization of the similarity matrix, we followed the original work to correct for multiple comparisons when determining the threshold using the false discovery rate (FDR) of 5%. Further detail of the applied methodology is provided in Supplementary Figure S1.

After the individual network was constructed for each subject, small-worldness σ (Sigma) was obtained based on the network properties computed with the Brain Connectivity Toolbox [38]. The two network properties, normalized path length (L) and the normalized clustering coefficient (CC) were first computed as the ratio of the L and CC between the network of interest and the random reference network, respectively. The σ was then calculated as the normalized CC divided by the normalized L. This corresponded to measuring how much the target network was clustered with respect to the shortest distance between nodes when compared to the random reference networks. A function from the MATLAB package GRETNA [39] was used for generating twenty random networks with the same number of nodes, number of edges, and degree distribution as the target network.

### 2.4. Global-Scale Statistical Analysis

Statistical analyses and visualization of the results were performed with Python 3.6.9 using packages Pandas (v.0.25.1), Pingouin (v.0.3.8), Matplotlib (v.3.1.1), and Seaborn (v.0.11.0). An independent t-test was performed to compare the subject characteristics including age, years of education, total GM volume, PAS score, and SAS score between the groups. A chi-square test was performed for the sex of the subjects.

To analyze the group difference in the small worldness, we performed the analysis of covariance (ANCOVA) of the metric σ controlling for the GM volume, which was found to be significantly different between the groups. Even though the level of education showed a group difference, it was not included as a covariate considering that covarying the years of education can lead to spurious results [40].

To specifically evaluate the relationship between the small worldness and the level of anhedonia in the two groups, partial correlation analysis controlling for the GM volume was performed between the σ and the two anhedonia scales. In the patient group, duration of illness and antipsychotic dosage were further controlled along with the GM volume. The partial correlation analysis was performed separately for the two groups, and the statistical difference of the correlation coefficient *r* between the two groups was evaluated with the *z*-test after the *r*-to-z Fisher transformation of the coefficient *r*. The two correlation analyses were corrected for multiple comparisons with the FDR method. To evaluate the group-specific correlation further, post hoc partial correlation analysis was performed for each group and was reported for regions with significant *z*-test results.

### 2.5. Local-Scale Statistical Analyses

**Figure 1:**
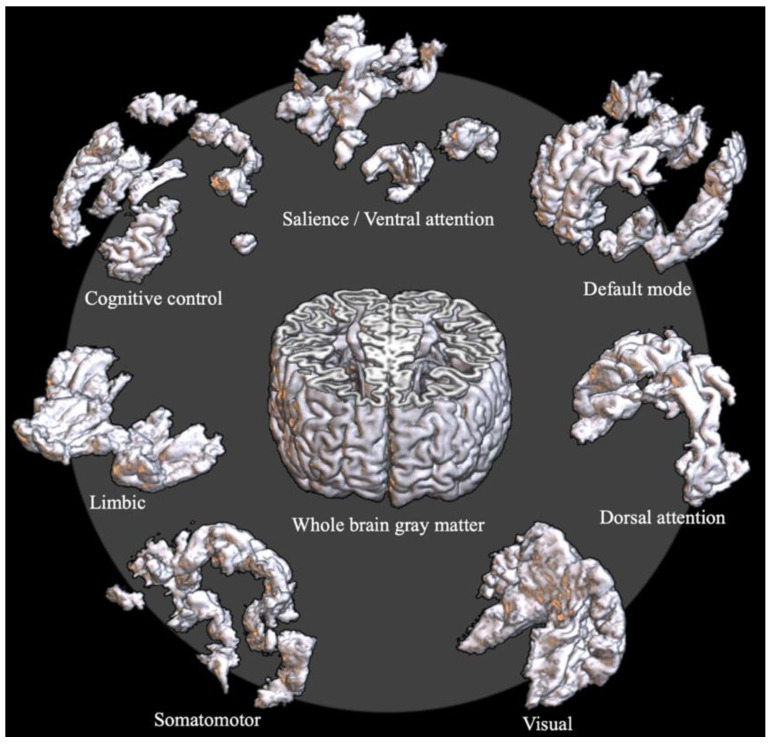
Exemplar T1 image of the segmented whole-brain grey matter (GM) volume and the intrinsic connectivity network (ICN)-level GM volumes. The ICN-level GM volumes were obtained by masking the whole-brain GM volume with the seven ICNs of the Schaefer 400 atlas.

To analyze network properties at a local scale, two standard space atlases were used. The first one was the Schaefer cortical parcellation atlas of 400 regions [41]. Each region of the atlas is labeled with one of the seven intrinsic connectivity networks (ICNs), such as the default mode network (DMN), cognitive control network (CCN), salience/ventral attention network (SVN), dorsal attention network (DAN), limbic network (LN), somatomotor network (SMN), and visual network (VN). The seven ICNs of the Schaefer atlas were used for the ICN-level local-scale analysis (Figure 1).

The second atlas used for the local scale analysis was the automated anatomical labeling (AAL) atlas [42]. The AAL includes 116 parcellated volumes of cortical, subcortical, and cerebellar regions in the standard MNI space. The AAL regions were used for the regional-level local-scale analysis.

For the registration of these volumes from the MNI space to individual T1 images, *applywarp* function of the FSL was used with the nonlinear warp field obtained during the anatomical processing step. By masking the regions from the two atlases, we produced 7 and 116 local-scale volumes per subject, respectively.

The individual network construction was performed to each local-scale volume with an identical method applied to the global-scale network construction. Since the individual network constructed from a very small volume may affect the robustness of the result, we omitted the regions with the average number of nodes across the subjects less than 30 from further statistical analyses. The list of omitted AAL regions is provided in Supplementary Table S1. Network metrics of each AAL region were computed with the same procedure as the global scale per subject.

The ANCOVA and the group comparison of the partial correlations were also performed for the local-scale network metrics. Each local-scale statistical analysis was corrected for the multiple comparisons with the FDR method based on the number of statistical tests performed.

## 3. Results

### 3.1. Subject Demographics and Scale Measurements

**Figure 2:**
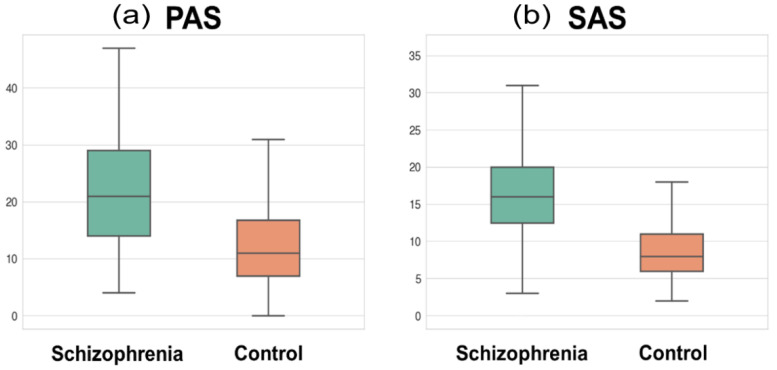
Box plot of (**a**) the physical anhedonia scale (PAS) and (**b**) social anhedonia scale (SAS) in the schizophrenia and control groups. The whiskers denote the farthest data from the box within the 1.5 inter-quartile range.

The subject demographics and scale measurements are presented in Table 1. The age and sex showed no statistical difference between patients and controls, while the number of education years was significantly shorter in patients than in controls (*t* = −9.47, *p* < 0.001). The total GM volume was significantly smaller in patients than in controls. The PAS and SAS scores showed significant group differences, indicating that patients had a higher level of physical and social anhedonia than controls (*t* = 8.66, *p* < 0.001; *t* = 8.44, *p* < 0.001, respectively; Figure 2). The mean chlorpromazine-equivalent dosage of antipsychotics was 419.8 ± 337.3 mg (median: 329.0) and the mean duration of illness was 9.7 ± 7.5 years (median: 8.0).

### 3.2. Global Network’s Small Worldness

The group comparison of the small-worldness metric σ controlling for the GM volume showed significant group difference (*F*_1277_ = 4.87, *p* = 0.028), whereas the comparison of the correlation coefficients between the two groups showed no statistical significance with the PAS score (*z* = 0.44, *p*-FDR = 0.662) and SAS score (*z* = 0.99, *p*-FDR = 0.640).

### 3.3. Network’s Small-World Metric at the Local Scale

The ANCOVA results of the network’s small-worldness metric of the ICN-level local-scale analysis are provided in Table 2. The significant regions included the DMN (*F*_1277_ = 11.03, *p*-FDR = 0.002), SVN (*F*_1277_ = 20.38, *p*-FDR < 0.001), and VN (*F*_1277_ = 15.10, *p*-FDR < 0.001). The ANCOVA of the AAL regional-level local-scale analysis showed no significant result.

**Figure 3:**
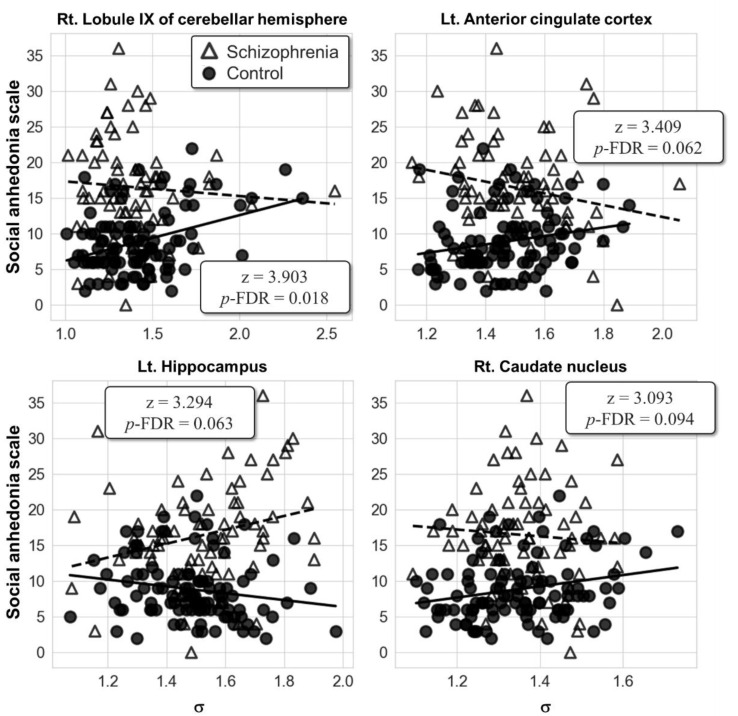
Scatter plots showing the relationships of the social anhedonia scale (SAS) score with the small-worldness metric σ from the results of the automated anatomical labeling (AAL)-level local-scale analysis. Abbreviation: Lt., left; Rt., right; FDR, false discovery rate.

No significant result was found in the group comparison of the partial correlation for the ICN-level local-scale analysis. However, the AAL-level local-scale analysis showed that the correlations between the SAS score and the right lobule IX of the cerebellar hemisphere (AAL index 106; *z* = 3.90, *p*-FDR = 0.018) were significantly different between the two groups (Figure 3). In the right lobule IX of the cerebellar hemisphere, the post hoc partial correlation analysis of each group resulted in the positive correlation between the SAS score and the σ in the control group (*r* = 0.36, *p* < 0.001), but not in the patient group. It is cautiously reported that the left ACC (AAL index 31; *z* = 3.41, *p*-FDR = 0.062), left hippocampus (AAL index 37; *z* = 3.29, *p*-FDR = 0.063), and right caudate nucleus (AAL index 72; *z* = 3.09, *p*-FDR = 0.094) fell into the trend of statistical significance with corrected *p*-FDR under 0.1. No significant correlation between the PAS score and the σ of the AAL regions was found in both groups.

## 4. Discussion

In this study, the small-world property of the GM network in patients with schizophrenia was studied with respect to the level of anhedonia. In the global-scale analysis, the level of small worldness was different between patients with schizophrenia and healthy controls. This group difference of the small-world property was also found in the ICN-level local-scale analysis. The DMN, SVN, and VN were the ICNs that showed a decrement in the GM network’s small worldness. From the AAL regional-level local-scale analysis, group comparisons of the correlation coefficients between the anhedonia scales and small worldness revealed that the right lobule IX of the cerebellar hemisphere showed different patterns of correlation in schizophrenia. The findings demonstrate that the GM network in schizophrenia may be characterized by the reduced small-world property at the global scale and the ICN-level local scale, and the small-world property may be aberrantly correlated with the level of social anhedonia at the AAL regional-level local scale.

The global-scale network analysis revealed a group average difference in the small worldness. The patient group showed a lower average σ than the control group, suggesting that patients with schizophrenia have decreased level of network efficiency in the GM network. Reduced small-world property in patients with schizophrenia is consistent with a number of previous findings that have reported the same phenomenon in the functional network, using electroencephalography [43,44] or functional MRI [24]. It can also be proposed that the GM network exhibits decreased small-world property in patients with schizophrenia from the group comparison result. These previous studies differ from our study in the modalities used and in the definition of the graph nodes because the nodes were defined adaptively to each subject in our study.

The group average difference of the small-world property was also apparent in the ICN-level local-scale analysis. The DMN, SVN, and VN have shown significantly reduced small worldness in the patient group when compared to the control group. Involvement of the structural and functional abnormality of the DMN has been thought to be one of the most important candidates for the neural basis of schizophrenia [45,46,47]. This abnormality of the DMN is thought to be related to various domains of the illness, including the level of positive symptoms [48], social functioning [49,50], and the long-term clinical outcome [51]. Specifically, there exists evidence that the dysfunction of resting-state DMN functional connectivity is associated with both physical and social anhedonia [52,53]. Structural abnormality of the DMN is also suggested to be related to the level of physical anhedonia [18].

Another ICN that showed reduced small-world property of the GM network in schizophrenia was the SVN. The SVN is thought to take a role in the pathophysiology of the illness along with the DMN [54,55]. The negative symptoms, such as amotivation, have been suggested to be related to the SVN via the salience network–midbrain abnormality leading to deficits in adequate reward processing in schizophrenia [56,57]. The VN was the last ICN that showed significantly reduced small-world property in the ICN-level local-scale analysis. The VN is responsible for visual memory processing, which is possibly related to anhedonia in schizophrenia [58]. Although the result of the ANCOVA from ICN-level local-scale analysis suggests that the small-world property of the GM network in schizophrenia may be reduced in the ICNs related to the negative symptoms, the significant relationship between anhedonia and small worldness of each ICN was not identified by the correlation analysis. This suggests that the GM network small-world topology cannot be said to carry a direct connection with anhedonia in the global and the ICN-level local scale. However, the connection with anhedonia was found in a more regional approach of the GM network topology.

The relationship between anhedonia and the small-world property of the GM network was revealed in the AAL regional-level local-scale analysis. Specifically, the group comparison of the partial correlations of σ and SAS score resulted in the different correlation pattern between the two groups in the right lobule IX of the cerebellar hemisphere. This result demonstrates that there exists an abnormal relationship between the small worldness of the GM network and the level of social anhedonia in the cerebellar hemisphere in patients with schizophrenia. Specifically, the SAS score correlated positively with the small worldness in the control group, whereas no relationship was found in the patient group. This result implies that patients with schizophrenia may show blunted response to the network topology of the cerebellar lobule with respect to the level of social anhedonia, unlike in healthy individuals. Another point to be noted from the result is that no significant correlation was found between the GM topology and the PAS score. Given the neuroimaging evidence that physical anhedonia and social anhedonia have separate neural correlates [52], and that the cerebellum is involved in social cognition [59,60], the GM topology of the cerebellar lobule seems to be related to social anhedonia rather than physical anhedonia.

The role of the cerebellum as the neural basis of schizophrenia has been suggested by the *dysmetria of thought* hypothesis, which hypothesizes that the cerebellar hemisphere regulates the rhythm or rate of motor movement and cognitive processes [61,62]. The hypothesis is supported by neuroimaging studies that show the relationship between cerebellar dysfunction and the various symptoms of schizophrenia [63,64,65]. Involvement of cerebellar abnormality in negative symptoms has also been found in functional connectivity studies [66]. This cerebellar abnormality might further be related to the disruption of the functional connectivity with the DMN, which has shown decrement in the GM network’s small worldness in the ICN-level local-scale analysis [67]. Our result implies that the GM network abnormality of the cerebellum may underlie the pathophysiology of social anhedonia in schizophrenia.

It is cautiously noted that the relationship between the small worldness of the GM network and the SAS score showed abnormality within the ACC, hippocampus, and caudate nucleus in a trend to the statistical significance. Interestingly, these three regions are known to be the components of the reward system in the human brain [10]. A large body of evidence has been reported regarding the relationship between the reward system dysfunctions and anhedonia [11]. In particular, the ACC is responsible for computing the efforts related to the expected reward, whereas the hippocampus and caudate nucleus take part in the prediction of the reward [68]. The results indicate a possibility that altered GM topology within the reward system may underlie the pathophysiology of social anhedonia via dysfunction of effort computation and reward prediction.

In sum, the results from our study indicate two main findings. (1) The small-world property of the GM network was reduced in patients with schizophrenia at the global-, DMN-, SVN-, and VN-level scales. (2) Alteration of the small worldness of the GM network in the cerebellar hemisphere, and possibly in the reward system, was related to social anhedonia in patients with schizophrenia. Considering that the small-world property represents the cost-efficient topology between the nodes of a network [26], it can be interpreted that patients with schizophrenia might have inefficiency of the cortical network topology. Anhedonia is a symptom arising from the complex interaction of specific functional impairments including the hedonic response, reward anticipation, emotion processing, incentive/effort calculation, and memory update [68]. The inefficient network structure of the brain may lead to a disruptive effect on this complex interaction, resulting in anhedonia. Altered correlation in the cerebellum and the reward system supports this idea and suggests a loss of intact communication between the hedonic experience, reward anticipation, and effort computation being related to the level of social anhedonia.

One thing that can be noted from the subject demographics is that the PAS and SAS scores fell below the midpoint of the scale in both groups. There is a possibility that these scales could not capture an aspect of anhedonia related to anticipatory reward as sensitively as the clinical assessment interview for negative symptoms (CAINS) [69]. Including other psychometric scales that can capture various features of anhedonia and related symptoms in the assessment and statistical analysis would make more detailed interpretation possible and valid in the future. Still, our findings provide a new insight to the neuroimaging community regarding anhedonia and GM network topology, which can potentially help find the neural basis and clinical biomarker of anhedonia in schizophrenia.

There are some limitations of the study that should be noted. First, the level of depressive symptoms was not assessed. Since anhedonia can also be a prominent feature in depressive episodes [70], the results would have been even more valid if the level of depression was statistically controlled. Second, while the PAS was collected from all participants, the SAS was evaluated only in some of the participants. If the SAS was obtained from all participants, the results would have had more statistical power. Lastly, some of the regions known to be possibly related to negative symptoms in schizophrenia (e.g., the posterior cingulate gyrus, amygdala) have been omitted from the AAL regional-level local-scale analysis. Methodological improvement that can robustly construct a GM network with a small number of nodes may provide a new way to reveal further findings from the regional-level analysis in the future.

## 5. Conclusions

The present study sheds light on the network perspective of the GM abnormality and its relationship with anhedonia in patients with schizophrenia. In the global-scale analysis, the patients showed reduced small-world property of the GM network. In the ICN-level local-scale analysis, the patients showed reduced small worldness in the DMN, SVN, and VN. The AAL regional-level local-scale analysis revealed that the left ACC, left hippocampus, and right lobule IX of the cerebellar hemisphere show an abnormal correlation with the level of social anhedonia in schizophrenia. These results indicate that reduced global and local GM network small-world property is present in schizophrenia, and the GM network abnormality of small worldness in the regions of the reward system may contribute to anhedonia in schizophrenia.

## Figures and Tables

**Table 1:** Group comparison of the subject demographics and scale measurements.

	Schizophrenia (n = 121)	Control (n = 160)	t	df	*p*
Age, years	34.5 ± 8.8	33.4 ± 6.8	1.06	224.18	0.291
Education, years	13.3 ± 2.2	16.2 ± 2.8	−9.47	273.69	<0.001
Sex, female/male	55/68	73/87	0.001	-	0.975
Grey matter volume, cm^3^	585.8 ± 60.5	600.0 ± 59.3	−1.97	259.92	0.049
Physical Anhedonia Scale score	21.5 ± 9.6	12.4 ± 7.5	8.66	227.02	<0.001
Social Anhedonia Scale score	16.4 ± 6.9	8.9 ± 4.4	8.44	120.37	<0.001

**Table 2:** Results from the group comparison of network’s small-worldness metric at the intrinsic connectivity network-level local scale analysis.

Intrinsic Connectivity Network	σ		*F*	*p*-unc	*p*-FDR
	Schizophrenia (n = 121)	Control (n = 160)			
Default mode network	1.298 ± 0.038	1.316 ± 0.044	11.03	0.001	0.002
Cognitive control network	1.227 ± 0.042	1.234 ± 0.037	2.57	0.110	0.128
Salience/ventral attention network	1.284 ± 0.050	1.312 ± 0.049	20.38	<0.001	<0.001
Dorsal attention network	1.207 ± 0.045	1.216 ± 0.043	3.04	0.083	0.116
Limbic network	1.326 ± 0.051	1.339 ± 0.050	3.41	0.066	0.116
Somatomotor network	1.294 ± 0.044	1.299 ± 0.042	0.89	0.346	0.346
Visual network	1.295 ± 0.053	1.320 ± 0.047	15.10	<0.001	<0.001

Abbreviation: unc, uncorrected; FDR, false discovery rate.

## Data Availability

The data presented in this study are available on request from the corresponding author. The data are not publicly available due to ethical restrictions.

## Supplementary Materials

The following are available online at https://www.mdpi.com/article/10.3390/jcm10071395/s1, Figure S1: Schematic illustration of the single-subject grey matter (GM) network construction method applied in the study, Table S1: The anatomical regions of the automated anatomical labelling atlas AAL omitted from the local scale statistical analysis.
