# Supplementary material for: Anhedonia Relates to the Altered Global and Local Grey Matter Network Properties in Schizophrenia
Source: J Clin Med. 2021 Mar 31;10(7):1395. doi: 10.3390/jcm10071395 (PMC8038049; doi:10.3390/jcm10071395)

Supplementary Table S1. The anatomical regions of the automated anatomical labelling atlas AAL omitted from the local scale statistical analysis

Index	Side	Region	Index	Side	Region
21	L.	Olfactory cortex	96	R.	Lobule III of cerebellar hemisphere
22	R.	Olfactory cortex	107	L.	Lobule X of cerebellar hemisphere
35	L.	Posterior cingulate gyrus	108	R.	Lobule X of cerebellar hemisphere
36	R.	Posterior cingulate gyrus	109		Lobule I, II of vermis
41	L.	Amygdala	110		Lobule III of vermis
42	R.	Amygdala	112		Lobule VI of vermis
75	L.	Pallidum	113		Lobule VII of vermis
76	R.	Pallidum	114		Lobule VIII of vermis
79	L.	Heschl’s gyrus	115		Lobule IX of vermis
80	R.	Heschl’s gyrus	116		Lobule X of vermis
95	L.	Lobule III of cerebellar hemisphere			

Supplementary Figure S1. Schematic illustration of the single-subject grey matter (GM) network construction method applied in the study.

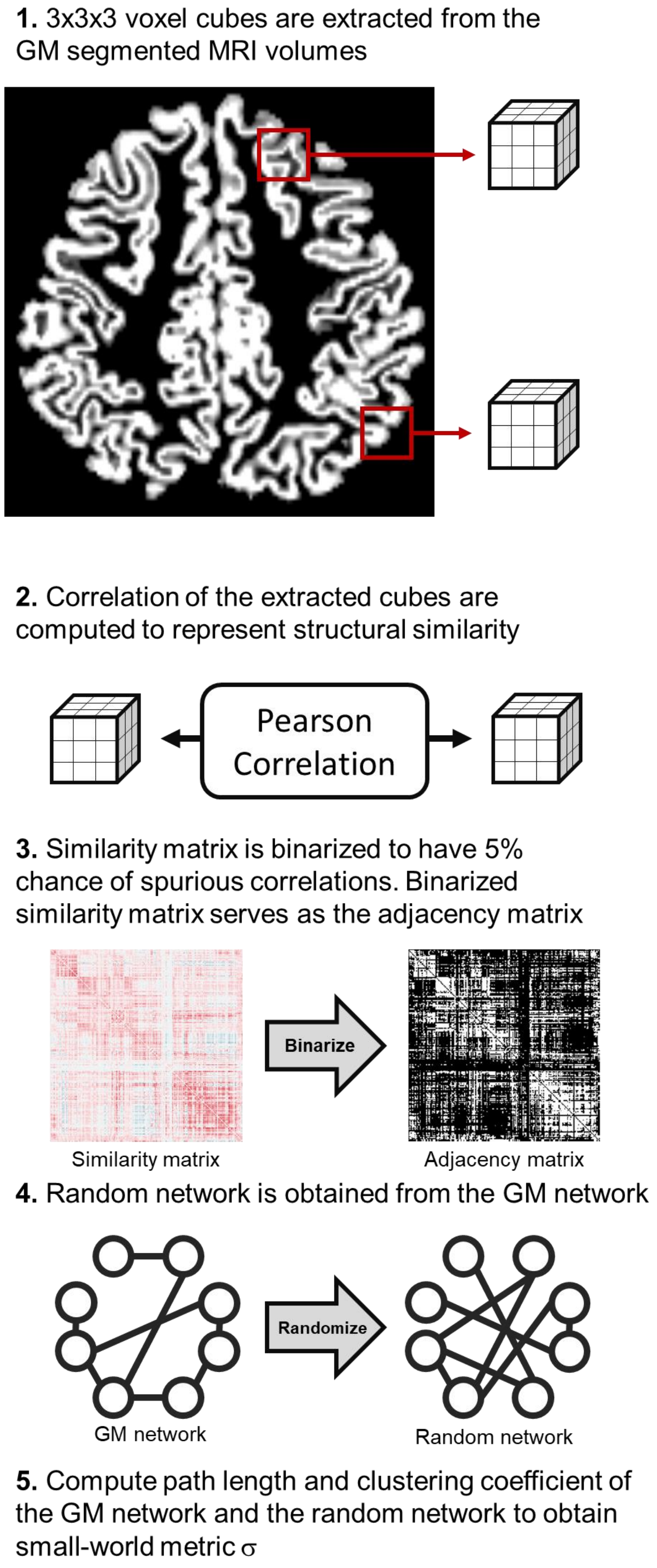

Supplement: Supplementary file 1 [file jcm-10-01395-s001.pdf]
